# Gender Differences in the Association between Moderate Alcohol Consumption and Hearing Threshold Shifts

**DOI:** 10.1038/s41598-017-02426-4

**Published:** 2017-05-19

**Authors:** Yuan-Yung Lin, Hsin-Chien Chen, Wen-Sen Lai, Li-Wei Wu, Chih-Hung Wang, Jih-Chin Lee, Tung-Wei Kao, Wei-Liang Chen

**Affiliations:** 1 Department of Otolaryngology–Head and Neck Surgery, Tri-Service General Hospital, National Defense Medical Center, Taipei, Taiwan Republic of China; 2 Graduate Institute of Medical Sciences, National Defense Medical Center, Taipei, Taiwan Republic of China; 3 Department of Otolaryngology-Head and Neck Surgery, Taichung Armed Forces General Hospital, Taichung, Taiwan Republic of China; 4 Department of Biological Science and Technology, Institute of Bioinformatics and Systems Biology, National Chiao Tung University, Hsinchu, Taiwan Republic of China; 5 Department of Family and Community Medicine, Division of Geriatric Medicine, Tri-Service General Hospital, Taipei, Taiwan Republic of China; 6 School of Medicine, National Defense Medical Center, Taipei, Taiwan Republic of China

## Abstract

Hearing loss is a global public health problem with a high prevalence, significantly impairing communication and leading to a decrease in the quality of life. The association between moderate alcohol consumption (MAC) and hearing impairment has been addressed in several studies with inconsistent results. The intent of our study is to clarify the correlation between MAC and the hearing threshold and further investigate the interplay between MAC and the hearing threshold categorized by gender. The study included 4,075 participants aged 20–69 years from the 1999–2004 data of National Health and Nutrition Examination Survey (NHANES). The associations among MAC, gender differences, and high-frequency and low-frequency hearing thresholds were analyzed. We found that current female drinkers with MAC tended to have lower hearing thresholds. There is a significant protective effect of MAC on hearing threshold shifts in the US adult population, especially in females. Our research was the first study to further indicate that there is a gender difference in the association between MAC and hearing impairment. In accordance with our results, if people drink, they should consume moderate rather than higher amounts, especially in women, which may result in a reduced risk of hearing loss.

## Introduction

Hearing loss is a highly prevalent sensory disorder and global public health problem. It significantly impairs communication and leads to a decrease in the quality of life because it negatively affects social interaction, leading to progressive isolation and withdrawal^1^. The widely known causes of hearing loss include aging, exposure to noise, ototoxic agents, inherent genetic determination, diabetes, hypertension, and cardiovascular disease^2^. By analyzing the possible contributing factors to hearing loss, many studies attempted to identify the possible modifiable factors that can potentially help to prevent or slow the development of hearing loss^3–7^. Notably, alcohol consumption seemed to have a protective effect on hearing impairment.

The most impressive effect of alcohol consumption is its protective association with cardiovascular disease. The accumulating evidence of consistent observational and mechanistic studies reveals that moderate alcohol consumption (MAC, 1 drink per day for women and 1–2 drinks per day for men) has beneficial effects on cardiovascular disease^8^. The plausible mechanisms about the protective effect of alcohol consumption on cardiovascular disease include increasing levels of plasma high-density lipoprotein (HDL) cholesterol^9, 10^, improving insulin sensitivity^11, 12^, the potential salutary effects of endothelial function^13, 14^, fibrinolysis/coagulation^15, 16^, and anti-inflammatory action^17, 18^.

The association between alcohol consumption and hearing impairment has also been reported in several research works. These studies appear to contradict one another; some of them noted a detrimental effect or positive association^19, 20^, some found a beneficial effect or negative association^4, 21–26^, and others observed no remarkable association^27–29^. Different study methods or purposes or different definitions of alcohol consumption might have been responsible for these inconsistent results. In general, the protective effect of alcohol consumption on hearing impairment was addressed, and the decreased protective effect or increased risk of hearing loss was noted regarding alcohol consumption at higher levels than moderate levels.

Gender differences in the consequences of alcohol use have been extensively studied. Women are more susceptible to the effects of alcohol consumption than men because women have higher blood alcohol concentrations after drinking equivalent doses of alcohol per kilogram of body weight^30–32^. Compared with men, women develop cirrhosis of the liver and more cognitive and motor impairment at lower levels of exposure to alcohol^33^. Gender differences in alcoholic cardiomyopathy, alcohol related behavioral or medical problems, and alcohol use and misuse in adults have also been reported^34^. Within the extensive research on alcohol consumption, comparatively little has focused on the gender differences in the association between MAC and hearing impairment. Among the studies indicating that MAC had a protective effect on hearing impairment, some did not find different effects between women and men^4, 21, 23^, while others did not analyze gender effects^22, 24^.

By studying the possible beneficial or risk factors for hearing impairment, we would be able to understand the etiologic factors of hearing loss, develop ideas for basic research, and make public health efforts for hearing preservation. Knowledge regarding the MAC, hearing impairment, and gender differences is rare. Given the theoretical positions taken for the study and the status of the field as briefly reviewed above, the aim of our study was to further clarify the interactions between MAC, hearing thresholds, and gender distribution using the National Health and Nutrition Examination Survey(NHANES) dataset.

## Results

### Characteristics of the study population

The present study included 4,075 participants obtained from the 1999–2004 NHANES dataset. The clinical and demographic characteristics of the study group were analyzed according to the patterns of alcohol consumption (Table 1). The current drinkers with MAC had a smaller waist circumference, lower fasting glucose levels, lower hearing threshold at low frequencies in the worse ear, lower frequencies of stroke, heart disease, and present use of ototoxic medication than lifetime abstainers and current drinkers with above moderate alcohol consumption (AMAC). The lifetime abstainers were younger, had lower uric acid and total cholesterol levels, lower hearing threshold at high frequencies in the worse ear, fewer males and non-Hispanic whites than those with the other three patterns of alcohol consumption.

**Table 1:** Characteristics of study participants.

Variables	Patterns of alcohol consumption				P value
	Lifetime abstainers (n = 582)	Former drinkers (n = 596)	Current drinkers with MAC (n = 2538)	Current drinkers with AMAC (n = 359)	
**Continuous variables, mean** ± **SD**					
Age (year)	40.38 ± 14.43	46.03 ± 12.44	40.53 ± 12.79	41.35 ± 12.40	<0.001
Waist circumference (cm)	96.65 ± 15.91	100.14 ± 16.31	95.51 ± 15.27	96.05 ± 14.05	<0.001
Uric acid (mg/dL)	4.93 ± 1.41	5.12 ± 1.46	5.24 ± 1.43	5.79 ± 1.53	<0.001
Fasting glucose (mg/dL)	102.67 ± 38.78	104.80 ± 38.60	99.71 ± 28.66	105.90 ± 50.81	0.029
Total cholesterol (mg/dL)	198.37 ± 41.86	210.67 ± 52.08	203.25 ± 43.33	208.64 ± 44.43	<0.001
Worse ear					
Low-PTA (dB)	15.70 ± 13.83	16.25 ± 11.74	13.60 ± 10.30	15.12 ± 11.15	<0.001
High-PTA (dB)	22.00 ± 17.87	28.69 ± 20.61	22.99 ± 18.17	26.56 ± 20.09	<0.001
Log low-PTA	1.08 ± 0.30	1.12 ± 0.28	1.04 ± 0.29	1.08 ± 0.29	<0.001
Log high-PTA	1.23 ± 0.36	1.35 ± 0.33	1.24 ± 0.33	1.30 ± 0.35	<0.001
**Categorical variables, n (%)**					
Male	149(25.6)	234(39.3)	1281(50.5)	216(60.2)	<0.001
Race					<0.001
Non-Hispanic white	197(33.8)	262(44.0)	1271(50.1)	192(53.5)	
Non-Hispanic black	164(28.2)	128(21.5)	470(18.5)	73(20.3)	
Other	221(38.0)	206(34.6)	797(31.4)	94(26.2)	
Ever had diagnosis					
Diabetes	59(10.3)	82(13.9)	122(4.9)	8(2.2)	<0.001
Heart disease	21(3.6)	36(6.1)	70(2.8)	12(3.3)	0.001
Stroke	10(1.7)	21(3.5)	30(1.2)	8(2.2)	0.001
Ototoxic medication (present use)	57(9.8)	81(13.6)	204(8.0)	31(8.6)	<0.001
Abnormal otoscopy	106(18.2)	128(21.5)	482(19.0)	76(21.2)	0.369
Abnormal tympanometry	73(14.0)	67(12.7)	236(10.1)	27(8.1)	0.012

Definition of abbreviations: MAC = moderate alcohol consumption. AMAC = above moderate alcohol consumption. SD = standard deviation. Low-PTA = Pure tone average at low frequencies. High-PTA = Pure tone average at high frequencies.

### Association between patterns of alcohol consumption and hearing thresholds

The results of patterns of alcohol consumption-based multiple linear regression analysis are presented in Table 2. Current drinkers with MAC tended to have lower hearing thresholds. The significant negative associations between MAC and the hearing thresholds, high-frequency pure-tone average (High-PTA) and low-frequency pure-tone average (Low-PTA), were noted in model 1–3. After adjusting with other covariates in model 3, the β coefficients of the High-PTA comparing the current drinkers with MAC to lifetime abstainers was −0.112 (p = 0.030); the β coefficients of the Low-PTA was −0.131 (p = 0.013).

**Table 2:** Regression coefficients of patterns of alcohol consumption for hearing threshold.

Variables		High-PTA				Low-PTA			
		Unadjusted	Model 1	Model 2	Model 3	Unadjusted	Model 1	Model 2	Model 3
Patterns of alcohol consumption									
Lifetime abstainers	β^*^ (95%CI)	Reference	Reference	Reference	Reference	Reference	Reference	Reference	Reference
	P value	—	—	—	—	—	—	—	—
Former drinkers	β (95%CI)	0.121 (0.083, 0.159)	−0.037(−0.142, 0.069)	−0.037(−0.143, 0.069)	−0.035(−0.142, 0.073)	0.035(0.002, 0.069)	−0.082(−0.192, 0.027)	−0.071(−0.181, 0.039)	−0.068(−0.178, 0.043)
	P value	<0.001	0.497	0.489	0.527	0.035	0.140	0.204	0.228
Current drinkers with MAC	β (95%CI)	0.011 (−0.019, 0.041)	−0.114 (−0.212, −0.015)	−0.116(−0.216, −0.017)	−0.112(−0.213, −0.011)	−0.048(−0.074, −0.022)	−0.146(−0.248, −0.044)	−0.133(−0.235, −0.030)	−0.131(−0.235, −0.027)
	P value	0.485	0.024	0.022	0.030	<0.001	0.005	0.012	0.013
Current drinkers with AMAC	β (95%CI)	0.140 (0.091, 0.188)	−0.069 (−0.180, 0.042)	−0.069(−0.181, 0.042)	−0.066(−0.181, 0.049)	0.032(−0.010, 0.074)	−0.107(−0.223, 0.008)	−0.102(−0.218, 0.013)	−0.111(−0.229, 0.007)
	P value	<0.001	0.223	0.224	0.261	0.140	0.068	0.082	0.065

Adjusted covariates: Model 1 = age, sex, race. Model 2 = Model 1 + (waist circumference, uric acid, fasting glucose, total cholesterol). Model 3 = Model 2 + (abnormal otoscopy, abnormal tympanometry, present use of ototoxic agents, history of diabetes, heart disease, stroke). Definition of abbreviations: MAC = moderate alcohol consumption, AMAC = above moderate alcohol consumption, High-PTA = Pure tone average at high frequencies, Low-PTA = Pure tone average at low frequencies. *β Coefficients can be interpreted as differences in hearing threshold comparing subjects in the other three patterns of alcohol consumption to those in the lifetime abstainers.

### Association between patterns of alcohol consumption and hearing thresholds in different genders

The relationships between patterns of alcohol consumption and hearing thresholds in different genders were analyzed and are presented in Table 3. Current female drinkers with MAC tended to have lower hearing thresholds. In model 1, model 2, and model 3, significant negative associations between MAC and hearing thresholds (High-PTA and Low-PTA) were observed. In model 3, the β coefficients of the High-PTA comparing the current female drinkers with MAC to lifetime abstainers was −0.154 (p = 0.013); the β coefficients of the Low-PTA was −0.150 (p = 0.034). However, no significant associations were observed in males.

**Table 3:** Regression coefficients of patterns of alcohol consumption and gender for hearing threshold

Variables	Patterns of alcohol consumption	High-PTA				Low-PTA			
		Men		Women		Men		Women	
		β (95%CI)	P value	β (95%CI)	P value	β (95%CI)	P value	β (95%CI)	P value
Model 1	Lifetime abstainers	Reference	—	Reference	—	Reference	—	Reference	—
	Former drinkers	0.074 (−0.132, 0.281)	0.479	−0.089 (−0.214, 0.036)	0.163	0.009 (−0.187, 0.206)	0.926	−0.123 (−0.265, 0.019)	0.089
	Current drinkers with MAC	−0.034 (−0.232, 0.165)	0.740	−0.134 (−0.248, −0.021)	0.020	−0.078 (−0.268, 0.111)	0.417	−0.168 (−0.296, −0.039)	0.011
	Current drinkers with AMAC	0.021 (−0.183, 0.225)	0.840	−0.138 (−0.325, 0.049)	0.148	−0.043 (−0.237, 0.152)	0.666	−0.076 (−0.288, 0.135)	0.477
Model 2	Lifetime abstainers	Reference	—	Reference	—	Reference	−	Reference	−
	Former drinkers	0.069 (−0.139, 0.276)	0.516	−0.093 (−0.219, 0.034)	0.150	0.018 (−0.178, 0.215)	0.854	−0.110 (−0.254, 0.033)	0.131
	Current drinkers with MAC	−0.038 (−0.239, 0.162)	0.708	−0.139 (−0.253, −0.024)	0.018	−0.063 (−0.253, 0.127)	0.513	−0.154 (−0.285, −0.024)	0.020
	Current drinkers with AMAC	0.015 (−0.190, 0.220)	0.886	−0.142 (−0.332, 0.049)	0.144	−0.039 (−0.233, 0.156)	0.696	−0.071 (−0.287, 0.145)	0.520
Model 3	Lifetime abstainers	Reference	—	Reference	—	Reference	—	Reference	—
	Former drinkers	0.061 (−0.147, 0.269)	0.564	−0.118 (−0.251, 0.015)	0.083	0.005 (−0.191, 0.202)	0.958	−0.109 (−0.261, 0.042)	0.156
	Current drinkers with MAC	−0.052 (−0.254, 0.150)	0.614	−0.154 (−0.276, −0.032)	0.013	−0.083 (−0.274, 0.108)	0.395	−0.150 (−0.288, −0.012)	0.034
	Current drinkers with AMAC	0.000 (−0.208, 0.209)	0.999	−0.209 (−0.411, −0.008)	0.042	−0.068 (−0.265, 0.130)	0.501	−0.103 (−0.332, 0.125)	0.374

Adjusted covariates: Model 1 = age, sex, race. Model 2 = Model 1 + (waist circumference, uric acid, fasting glucose, total cholesterol). Model 3 = Model 2 + (abnormal otoscopy, abnormal tympanometry, present used of ototoxic agents, history of stroke, diabetes, heart disease). Definition of abbreviations: MAC = moderate alcohol consumption, AMAC = above moderate alcohol consumption, High-PTA = Pure tone average at high frequencies, Low-PTA = Pure tone average at low frequencies.

## Discussion

Based on analyzing the national representative sample of US adults, we found that current drinkers with MAC had significant negative associations regarding high-frequency and low-frequency hearing thresholds compared with lifetime abstainers but former drinkers and current drinkers with AMAC did not. That is, current drinkers with MAC had lower high-frequency and low-frequency hearing thresholds than lifetime abstainers. It is worth noting that this association was observed in female subjects but not in male. MAC seems to have a protective effect on hearing threshold shift only in women. To our best knowledge, ours was the first study to indicate a gender difference in the association between MAC and hearing impairment.

Several studies have suggested that alcohol consumption has a protective effect on hearing impairment. Compared to never drinkers, some studies reported that the protective effect was only noted in current drinkers with MAC (a U-shaped association was presented between alcohol consumption and hearing impairment with no risk for AMAC and lower risk for MAC)^4, 21, 22, 24^, and other studies report that the protective effect was observed in current drinkers with all categories, even above average or higher levels^25, 26^. The results of the Japanese epidemiological cross-sectional study with 496 subjects by Itoh *et al*.^22^ and the Blue Mountains Hearing Study with 2956 subjects in Australia by Gopinath *et al*.^24^ showed the protective effect of MAC. However, Itoh *et al*. did not differentiate high and low frequency hearing loss, Gopinath *et al*. only found significance in low frequency hearing loss, and these two studies did not analyze the gender effect. In another two cross-sectional studies by Popelka *et al*. in America^21^ and Fransen *et al*. in Europe^4^, they found a protective effect of MAC in both low and high frequency hearing loss, which were consistent with our results. However, the gender differences were not observed in their analysis. Our findings extended the scanty literature about MAC and hearing loss and further indicated the gender differences. Further larger studies or prospective surveys are still needed to explore the gender differences in the protective effect of MAC on hearing impairment more clearly.

The protective effects of alcohol consumption on hearing impairment may occur through several possible pathways or mechanisms. First, as far as we know, cardiovascular disease is associated with hearing loss^2^. Some studies proposed that the beneficial cardiovascular effects of alcohol consumption lead to the decreased risks of hearing loss^4, 24, 25^. Second, alcohol consumption may increase plasma HDL cholesterol concentrations^8–10^, contribute to better endothelial function^13, 14^, and reduce coagulation^15, 16^, which contribute to an optimal cochlear circulation and an intensifying anti-atherosclerotic condition and result in decreased hearing impairment. Third, alcohol consumption had anti-inflammatory action and neuroprotection^17, 18, 35^ that may help to enhance cellular survival of the cochlea. Finally, a U-shape association between alcohol consumption and hearing impairment was reported and the lack of enhanced benefits of higher levels of alcohol consumption or AMAC on hearing impairment is likely due to the adverse effects on other risk factors for hearing loss^4, 21, 22, 24^.

The tonotopic distribution of the cochlear hair cells leads to the base part of the cochlea responding to high frequencies and the apical part to low frequencies. We found a protective effect of MAC on the hearing threshold shift in both low and high frequency. It indicated this effect was influencing the whole cochlea. It was consistent with the strial hearing loss proposed by Schuknecht^36^, which is characterized by histopathologic findings with atrophy of the stria vascularis in the whole cochlea and by clinical results of decreased hearing sensitivity across the whole frequency spectrum. In other words, the protective effect of MAC may be related to the involvement of the stria vascularis, affecting the microvasculature of the cochlea. Notably, this is consistent with the possible mechanism about the protective effects of alcohol consumption that we described in the second point above; further study is required to investigate whether the stria vascularis of the whole cochlea is affected by alcohol consumption.

Lifestyle variables may also be confounding factors of the hearing protective effects between current drinkers with MAC and never drinkers. Emerging studies have reported that never drinkers had a higher body mass index, less regular exercise, lower vegetable consumption, and higher fat intake comparing with all drinkers^37^. Current drinkers with MAC were observed to pay more attention to their health using preventive care or improving their health behaviors than never and heavy drinkers^38^. Other confounding factors from MAC, such as socioeconomic status, have also been suggested^39^.

The gender differences in the association of MAC and hearing threshold shifts is likely due to innate biological differences. Women are more susceptible to the effects of alcohol consumption than men because women have higher blood alcohol concentrations after drinking equivalent doses of alcohol per kilogram of body weight^30–32^. There are several differences between men and women in the pharmacokinetics of alcohol. Women have lower gastric alcohol dehydrogenase; thus, the first stage of alcohol metabolism is decreased and more alcohol is absorbed into the blood stream^30, 31^. Women have a higher percentage of body fat^32^ and a lower percentage of body water than men^40^; thus, each drink is more concentrated in women’s blood circulation. Therefore, gender differences should always be considered when studying the effects of alcohol consumption.

There are some limitations in our study. First, causality cannot be claimed because of the cross-sectional design of our study. Second, we could not exclude potential recall bias for information about the frequency and quantity of past and current alcohol consumption and other personal and medical history data. Third, the questions in the Alcohol Use Questionnaire are about drinking alcoholic beverages, including liquor, wine, beer, and any other type of alcoholic beverage. The types of alcoholic beverages cannot be differentiated in our dataset. Fourth, there was no information about genetic or congenital hearing impairment in the NHANES data.

## Conclusions

Our study highlighted that there is a significant protective effect of MAC on hearing threshold shifts in the US adult population, especially in female individuals. This study may lead to a better understanding of the interplay between gender differences, MAC, and hearing threshold shifts. If people drink, they should consume moderate rather than higher amounts, especially in women, which may result in a reduced risk of hearing loss. Further research is warranted to clarify the benefit of MAC on hearing threshold shifts and gender differences.

## Methods

### Description of NHANES and study population

NHANES is a special cross-sectional survey that combines personal interviews, standardized physical examinations, and numerous laboratory studies. It was directed by the Centers for Disease Control and Prevention’s National Center for Health Statistics and designed to gather information about the health, nutritional status, and health behaviors of the non-institutionalized civilian resident population of the United States. Starting in 1999, it became an ongoing annual survey; the data sets have been released every two years on the NHANES website.

We limited our study subjects to 1999–2004 NHANES individuals who had completed both the personal questionnaires about alcohol consumption and audiometric examinations. Individuals were excluded if they had current or past occupational noise exposure and if their relevant data were incomplete. The final analytic data sets included 4,075 subjects aged 20–69 years, including demographic data, the results of physical investigations and laboratory tests, questionnaire information, and audiometric examinations.

### Audiometric measurements

According to the 1999–2004 NHANES protocol, half of the included participants were randomly arranged for audiometric examinations after household personal interview and medical examinations. The participants were not included if they could not remove their hearing aids or bear with the headphones. Audiometric measurement was performed in a mobile examination center’s sound-isolated room by technicians who had been trained by a qualified audiologist from the National Institute for Occupational Safety and Health. Audiometric equipment included an interacoustic model AD226 audiometer with EARTone 3 A insert earphones and standard TDH-39P headphone. Each ear was tested from 500 to 8000 Hz with an intensity-range of −10 to 120 dB to determine the air-conduction pure tone hearing thresholds. The average of pure tone hearing thresholds at 3,000, 4,000, 6,000, and 8,000 Hz was defined as the high-frequency pure-tone average (High-PTA). The average of pure tone hearing thresholds at 500, 1,000, and 2,000 Hz was defined as the low-frequency pure-tone average (Low-PTA). The worse ear with the lower pure-tone average was chosen for the regression analysis^41^.

### Definition of patterns of alcohol consumption

The Alcohol Use Questionnaire (ALQ) in NHANES was designed to collect information about the frequency and quantity of past and current alcohol consumption. The definition of a drink was an ounce of liquor, a 4-ounce glass of wine, or a 12-ounce beer. The variable of drinks per day (dkspd) was calculated by the ALQ data^42^. We classified individuals into the following four groups^42–44^: (I) lifetime abstainers- < 12 drinks in entire life; (II) former drinkers− ≧ 12 drinks in the past but none during the past 12 months; (III) current drinkers with MAC- < 0 to ≦2 dkspd in men and <0 to ≦ 1 dkspd in women during the past 12 months; and (IV) current drinkers with AMAC- > 2 dkspd in men and >1 dkspd in women during the past 12 months.

### Assessment of covariates

Demographic data, including age, gender, race, and medical history were collected. Race was classified as non-Hispanic white, non-Hispanic black, or other. Individuals were considered to have diabetes if there was a physician’s diagnosis by self-report medical history, random and fasting glucose level greater than or equal to 200 and 126, respectively, and/or the use of diabetic medications. Heart disease was considered positive if an individual had a diagnosis of or had experienced congestive heart failure, coronary artery disease, myocardial infarction or angina. Stroke was ascertained by self-reports. The present use of ototoxic medication, including non-steroidal anti-inflammatory drugs, anticancer drugs, aminoglycoside, or loop diuretics was defined by self-reports. Waist circumference was determined at the top of the hip bone after breathing out normally to the nearest 0.1 cm. Abnormal otoscopy was recorded if there were abnormalities in screening otoscopic examination of the eardrums and ear canals before the audiometric measurement. Tympanometry was determined as abnormal if the compliance was less than or equal to 0.3 ml or middle ear peak pressure was less than or equal to −150 daPa using an audiometric tympanometer. Measurement of serum uric acid was determined using a Hitachi 737 analyzer. The level of total cholesterol was measured using a Hitachi 704 analyzer. The level of serum fasting glucose was measured using the Cobas Mira assay. All associated information about biospecimen collection and processing protocols is available on the NHANES website.

### Statistical analysis

SPSS (version 18.0 for Windows; SPSS, Inc., Chicago, IL, USA) was used to perform the statistical analyses. The significant differences were defined as two-sided p-values < 0.05. Covariates, including age, waist circumference, uric acid, fasting glucose, and total cholesterol were managed as continuous variables and presented as the mean value with standard deviation. Other covariates, such as gender and race were treated as categorical variables and expressed as numbers with percentages. Logarithmic transformation was performed for the values of the PTA hearing thresholds to approximate a normal distribution. Our study used patterns of alcohol consumption-base analysis by dividing participants into four groups with the lifetime abstainers as the referent group. The linear regression model was used to evaluate the effect of patterns of alcohol consumption on the hearing threshold. A multiple-model analysis was performed for adjustments of covariates^41^. First, age, gender, and race were adjusted in model 1. Model 1 subsequently adjusted for waist circumference, uric acid, fasting glucose, and total cholesterol was model 2. Model 2 further adjusted for abnormal otoscopy and tympanometry, current use of ototoxic medication, history of diabetes, heart disease and stroke was model 3.

### Ethics statement

The Research Ethics Review Board of National Center for Health Statistics approved the NHANES study and underwent annual review. All eligible subjects provided their written informed consent before participating the study. Because de-identified data were used in our study, it was exempt from IRB review.
